# Alteration of Plasma Glutamate and Glutamine Levels in Children with High-Functioning Autism

**DOI:** 10.1371/journal.pone.0025340

**Published:** 2011-10-05

**Authors:** Chie Shimmura, Shiro Suda, Kenji J. Tsuchiya, Kenji Hashimoto, Koji Ohno, Hideo Matsuzaki, Keiko Iwata, Kaori Matsumoto, Tomoyasu Wakuda, Yosuke Kameno, Katsuaki Suzuki, Masatsugu Tsujii, Kazuhiko Nakamura, Nori Takei, Norio Mori

**Affiliations:** 1 Department of Psychiatry and Neurology, Hamamatsu University School of Medicine, Hamamatsu, Japan; 2 Research Center for Child Mental Development, Hamamatsu University School of Medicine, Hamamatsu, Japan; 3 Department of Anatomy and Neuroscience, Hamamatsu University School of Medicine, Hamamatsu, Japan; 4 Center of Forensic Mental Health, Chiba University, Chiba, Japan; 5 Faculty of Sociology, Chukyo University, Nagoya, Japan; 6 Division of Psychological Medicine, Institute of Psychiatry, London, United Kingdom; Biological Research Center of the Hungarian Academy of Sciences, Hungary

## Abstract

**Background:**

It has recently been hypothesized that hyperglutamatergia in the brain is involved in the pathophysiology of autism. However, there is no conclusive evidence of the validity of this hypothesis. As peripheral glutamate/glutamine levels have been reported to be correlated with those of the central nervous system, the authors examined whether the levels of 25 amino acids, including glutamate and glutamine, in the platelet-poor plasma of drug-naïve, male children with high-functioning autism (HFA) would be altered compared with those of normal controls.

**Methodology/Principal Findings:**

Plasma levels of 25 amino acids in male children (N = 23) with HFA and normally developed healthy male controls (N = 22) were determined using high-performance liquid chromatography. Multiple testing was allowed for in the analyses. Compared with the normal control group, the HFA group had higher levels of plasma glutamate and lower levels of plasma glutamine. No significant group difference was found in the remaining 23 amino acids. The effect size (Cohen's *d*) for glutamate and glutamine was large: 1.13 and 1.36, respectively. Using discriminant analysis with logistic regression, the two values of plasma glutamate and glutamine were shown to well-differentiate the HFA group from the control group; the rate of correct classification was 91%.

**Conclusions/Significance:**

The present study suggests that plasma glutamate and glutamine levels can serve as a diagnostic tool for the early detection of autism, especially normal IQ autism. These findings indicate that glutamatergic abnormalities in the brain may be associated with the pathobiology of autism.

## Introduction

Autism is a neurodevelopmental disorder that affects 1–2 in 100 children, according to recent data on the broad array of autism spectrum disorders (ASD) [1]. The disorder is characterized by severe and sustained impairment of social interaction, abnormalities in communication, and patterns of repetitive behaviors and restricted interest [2]. Although genetic predisposition and environmental contributors have been implicated in the pathophysiology of autism, the precise mechanisms underlying the pathophysiology of this disorder remain unknown [3] and there are no established methods of prevention or cure. Receiving diagnosis at an early stage of development could contribute to the acquisition of optimized coping strategies for both patients and their families. However, while diagnosis of autism is based solely on behavioral abnormalities, such abnormalities are often overlooked in early life, even among professionals involved in pediatric healthcare [4]. Therefore, it remains necessary to establish a reliable hallmark that could contribute to an early and more precise autism diagnosis.

Glutamate, the major excitatory neurotransmitter, is highly concentrated throughout the brain and is crucial to neuronal plasticity and the maintenance of cognitive functioning. However, excess glutamate has been shown to be a potent neurotoxin that leads to neuronal cell death [5], [6] and is deemed to play a role in the pathophysiology of some neuropsychiatric disorders [7]. Recently, a hyperglutamatergic hypothesis of autism was proposed [8], [9] based on evidence of hyperglutamatergia in the brain of individuals with autism. For instance, Fatemi et al. [10] have shown that levels of GAD 65 kDa and GAD 67 kDa proteins, both of which are involved in converting glutamate to GABA, are reduced in the brains of individuals with autism, resulting in increased levels of glutamate in the brain substrate. In addition, a neuroimaging magnetic resonance spectroscopy study by Page et al. (2006) has demonstrated that individuals with ASD have significantly higher concentrations of glutamate in the amygdala-hippocampal region than do healthy controls [11].

Although glutamate is thought not to readily cross the blood brain barrier [7], the level of glutamate in the blood is positively correlated with the cerebrospinal fluid (CSF) level of glutamate in humans [12], [13]. In fact, increased plasma levels of glutamate have been reported in some neuropsychiatric disorders, such as epilepsy [14], Alzheimer's disease [15] and amyotrophic lateral sclerosis [16], in which glutamate excitotoxicity is thought to play a role in the pathophysiology. Thus, the peripheral glutamate level can be postulated to reflect the glutamate level in the brain *per se*. In our previous study, we found that the serum level of glutamate was significantly higher in young adults with autism than in adult controls [17], suggesting that individuals with this condition may have a high concentration of glutamate in the brain. However, the results reported in the literature are contradictory, with some studies showing no association between serum glutamate and autism [18], [19]. Such inconsistencies could be due to differences among the samples studied. In fact, there has been considerable variation among the previous studies [17], [18], [19], [20] in terms of age, gender, IQ, and use of psychoactive medication, as well as in the methods of blood sample collection (e.g., serum, platelet-rich plasma, or platelet-poor plasma).

Because autism is a heterogeneous disorder [21], the selection of study samples is likely to affect the results. In order to make our study sample as homogeneous as possible and to render the findings easily interpretable (and comparable), we limited the inclusion of subjects to drug-naïve, male children with high-functioning autism (HFA), and we chose platelet-poor plasma for the blood sample collection. The rationale for these choices was as follows. First, the findings of a sample of adults may merely reflect glutamate levels related to the duration of illness, and may therefore represent secondary changes in response to some compensatory process. Therefore, it is preferable to examine a sample of children with the disorder, since children are less likely to be influenced by the disease duration. Second, it is well documented that 40–62% of individuals with autism have some intellectual impairment [22]. Controlling for the Intelligence Quotient (IQ) or excluding those individuals with autism who have intellectual disabilities could eliminate factors that are related to general intellectual impairments but unrelated to autism. Third, because sex differences in autism have been reported [23], [24], [25], we chose to examine only male subjects, among whom autism is more prevalent. Fourth, since neurotransmitter systems are extremely sensitive to psychoactive medications, we recruited only drug-naïve individuals with HFA. Finally, some amino acids, such as glutamate and glutamine, are contained in blood platelets [26]; thus, when amino acids are examined in peripheral blood, platelet-poor plasma samples could yield more informative data than serum samples.

We therefore performed a high-performance liquid chromatography (HPLC) analysis of glutamate levels in the platelet-poor plasma of drug-naïve, male children with HFA. Since amino acids exert various influences on one another in the process of amino acid synthesis/degradation, any changes in plasma glutamate levels could be attributed to changes in other amino acids or in related compounds such as urea and taurine. Therefore, we comprehensively analyzed a total of 41 amino acids and amino acid-related compounds (for brevity, hereafter amino acids), including glutamate, in the plasma of male, drug-naïve children with autism. As the levels of 16 amino acids (see Materials and Methods below) were found to be undetectable by HPLC, we ultimately investigated the plasma levels of only 25 amino acids (see Table 1) in the individuals with autism and healthy controls. We hypothesized that plasma levels of glutamate, but not those of other amino acids, would be elevated in children with autism relative to those of healthy children.

**Table 1:** Plasma Levels of Amino Acids and related compounds in Control Subjects and Individuals with HFA.

Amino acid	Control	HFA	*p*-value
Alanine	326.1±61.6	300.3±55.0	0.145
α-Aminobutyric acid	18.8±3.8	18.7±5.4	0.971
Arginine	89.1±19.0	95.3±18.5	0.279
Asparagine	40.8±8.3	43.1±7.0	0.311
Citrulline	29.2±6.1	29.1±4.7	0.934
Cysteine	21.9±3.7	22.5±3.6	0.586
Glutamate	20.9±4.5	27.9±7.4	<0.002*
Glutamine	513.1±48.5	445.8±50.6	<0.0004**
Glycine	220.4±32.2	202.7±42.9	0.127
Histidine	73.1±6.4	76.6±7.9	0.120
Hydroxyproline	20.5±2.2	22.5±3.6	0.097
Isoleucine	53.6±11.5	62.2±14.5	0.033
Leucine	99.0±16.1	106.4±22.4	0.210
Lysine	155.3±28.5	164.2±32.5	0.332
Methionine	23.7±5.1	25.8±5.6	0.203
Ornithine	43.9±11.3	51.9±10.8	0.021
Phenylalanine	51.7±6.8	55.1±8.4	0.146
Proline	153.7±56.4	131.7±47.6	0.165
Serine	105.4±15.6	115.8±14.7	0.027
Taurine	33.4±5.5	37.8±7.9	0.036
Threonine	100.8±19.7	112.0±24.3	0.097
Tryptophan	44.8±5.6	47.3±6.4	0.167
Tyrosine	60.9±10.5	58.4±10.1	0.425
Urea	3976.3±818.7	3759.9±773.3	0.367
Valine	200.2±29.4	217.1±29.7	0.062

HFA: High-Functioning Autism.

Values are expressed as the mean ± SD.

Two-tailed unpaired *t*-test with the Bonferroni correction,

*p<0.002,

**p<0.0004.

## Results

**Figure 1:**
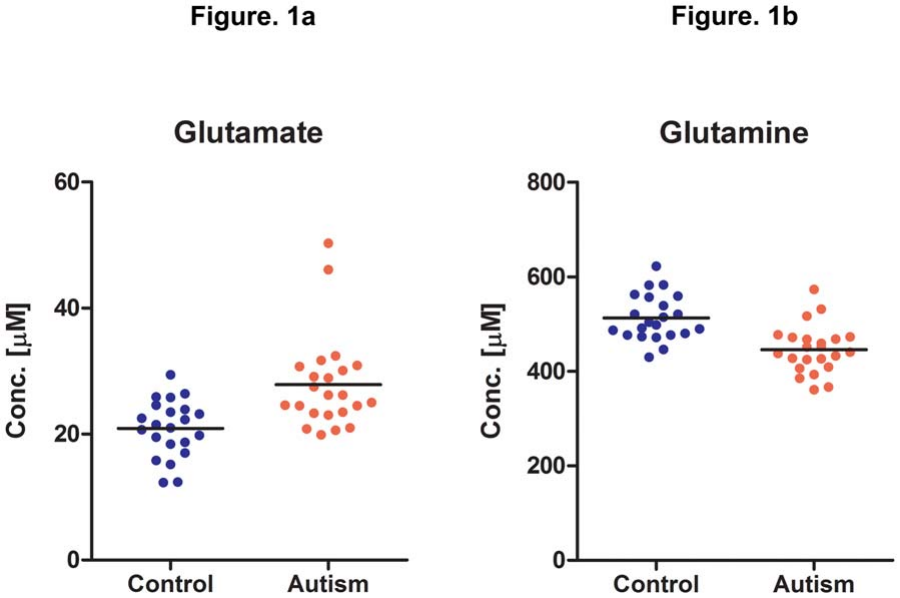
Plasma Levels of Glutamate and Glutamine in Control Subjects and Individuals with HFA. Fig. 1-a: Glutamate Levels; Fig. 1-b: Glutamine Levels. Plasma levels of glutamate and glutamine were determined using HPLC. Control subjects, N = 22; individuals with HFA, N = 23. *Left to right: t* = 3.77, *df* = 43, *p*<0.002; *t* = −4.55, *df* = 43, *p*<0.0004. The plasma glutamate level was significantly higher (*p*<0.002), and the plasma glutamine level was significantly lower (*p*<0.0004) in the HFA group than in the control group. When two autistic cases with extreme values were eliminated from the glutamate data, the results changed only negligibly; *p*<0.002.

There was no significant difference in age (*t* = 1.79, *df* = 43, *p* = 0.081), IQ (*t* = −2.00, *df* = 43, *p* = 0.052), BMI (body-mass index; *t* = 1.06, *df* = 43, *p* = 0.29), or plasma creatinine levels (*t* = 0.99, *df* = 43, *p* = 0.33) between the HFA and control groups (Table 2). Although non-significant, the marginal difference in age and IQ could not be overlooked, and was taken into account in subsequent analyses. The plasma levels of 25 amino acids were analyzed using HPLC, and are shown in Table 1. There were no significant intergroup differences in the amino acid plasma levels, with the exception of glutamate and glutamine (Table 1). Only the results for these two amino acids, i.e., glutamate and glutamine, met the criterion for statistical significance. As anticipated, the plasma level of glutamate was significantly higher in the HFA group (mean ± S.D. = 27.9±7.4 µM) than in the control group (mean ± S.D. = 20.9±4.5 µM) (*t* = 3.77, *df* = 43, *p*<0.002) (Fig. 1a); the mean difference was 7.0 µM (95% confidence interval [CI]: 3.3 to 10.7 µM), with a Cohen's *d* of 1.13. In contrast, the plasma level of glutamine in individuals with HFA (mean ± S.D. = 445.8±50.6 µM) was significantly lower than that of normal controls (mean ± S.D. = 513.1±48.5 µM) (*t* = −4.55, *df* = 43, *p*<0.0004) (Fig. 1b); the mean difference was −67.3 µM (95% CI: −97.2 to −37.5 µM), with an effect size of 1.36.

**Table 2:** Clinical Characteristics of Control Subjects and Individuals with HFA.

	Control (n = 22)	HFA (n = 23)	*p*-value
Age (years)	12.2±2.4 (9–17)	13.5±2.5 (8–17)	0.081
Full-scale IQ	108.1±14.6	99.6±13.5	0.052
BMI	17.8±2.1	18.6±3.3	0.29
Creatinine (mg/dl)	0.50±0.13	0.53±0.13	0.33
ADI-R			
Domain A score, social		18.3±5.2 (12–26)	
Domain BV score, communication		11.6±4.4 (3–21)	
Domain C score, stereotype		5.1±2.8 (1–9)	

BMI: body mass index.

ADIR: Autism Diagnostic Interview-Revised.

HFA: High-Functioning Autism.

Values are expressed as the mean ± SD.

Two-tailed unpaired *t*-test.

Since marginal differences in age and IQ were noted between the two groups, ANCOVA was used to adjust for these factors. When the measurement of the glutamate levels was adjusted for age and IQ, the difference remained significant (*p*<0.002 after adjustment for age; *p*<0.002 after adjustment for IQ). When the measurement of the glutamine levels was adjusted for age and IQ, the difference remained significant for IQ (*p*<0.0004), but became even more conspicuous after adjustment for age (*p*<0.0001).

Using discriminant analysis with logistic regression that included the four variables of age, IQ, and plasma glutamate and glutamine levels, the correct classification rate was found to be 91%. The sensitivity and specificity were 90% and 91%, respectively. The parameter estimates in this logistic regression model are shown in Table 3.

**Table 3:** Parameter Estimates in Discriminant Analysis with Logistic Regression: Coefficients Based on the Logit Model.

Variables	Coefficient	95%CI	*p*-value
Age (years)	0.88	−0.25–2.01	0.13
IQ	−0.07	−0.16–0.03	0.19
Glutamate	0.73	0.05–1.41	0.04
Glutamine	−0.09	−0.18–−0.004	0.04
Constant	23.52	−3.08–50.12	0.08

Discriminant analysis with logistic regression and four included variables: age, IQ, and plasma glutamate and glutamine levels; correct classification rate, 91%.

We then examined the correlations between plasma glutamate/glutamine levels and autism-related symptoms of three separate domains among individuals with HFA. There was no significant correlation between the glutamate/glutamine levels and the three subscores as assessed by the ADI-R.

## Discussion

We found that the platelet-poor plasma level of glutamate in children with HFA was significantly higher than that of normally developing children, which supports the hyperglutamatergic hypothesis of autism. In our previous study, we demonstrated an increased level of glutamate in young adults (18–26 years) with autism [17]. That finding, taken together with the results of the current study, suggests the presence of elevated glutamate in the plasma of autistic individuals early in life. Moreover, this elevation appears to be sustained over a long period of time, i.e., into adulthood. Thus, it appears likely that the plasma glutamate level could serve as a long-term stable screening tool for autism.

In addition, we found that the plasma level of glutamine was significantly lower in children with HFA than in the controls. This finding was not anticipated prior to the study, and the difference between the HFA and control groups was highly significant (*p*<0.0004). Thus, it is very unlikely that this difference between the two groups could be attributed to a chance finding. Of note in this context are the identical results of a previous study by Aldred et al. [20] showing reduced levels of glutamine together with elevated levels of glutamate in the plasma of individuals with autism. However, a general interpretation of that study—and more specifically, a direct comparison with the present study—is hampered by methodological differences. In the study by Aldred et al. [20], a smaller number of amino acids (n = 17) was examined compared with our study (n = 25); a looser criterion for statistical significance of *p*<0.05 was used (no account was taken of multiple testing); controls were chosen from those who had been admitted to a hospital due to physical problems and, thus, were more similar to autistic subjects in terms of poor health; the age range was wider and adults were included (4–29 years for the autism group); and platelet-rich, instead of platelet-poor, plasma was used, which rendered the present study using platelet-poor plasma advantageous in terms of the serological investigation of autism, as described above.

Due to the heterogeneity of autism [21], we attempted to recruit and analyze a relatively homogeneous group. We therefore restricted our study sample to the following: males, relatively young subjects (to minimize the factors related to the illness duration), only drug-naïve cases, and those individuals with a full-scale IQ of higher than 70 (i.e., HFA). Further, the amount of muscle mass in the body is important in examining glutamine and glutamate levels, because the skeletal muscle accounts for a major portion of glutamine synthesis. Failure to control for variation in the amount of body muscle would lead to unreliable findings. In this study, we assessed the plasma creatinine level, which reflects the quantity of muscle mass, and we also included BMI in our evaluation. We found no significant group differences in these variables, suggesting that variation in the skeletal muscle mass was unlikely to have confounded our results. However, we also performed additional ANCOVA, in which we adjusted for plasma creatinine levels and BMI. In these analyses, the significant glutamate and glutamine findings remained unchanged (*p*<0.002 for all comparisons).

In this study, substantial differences between children with HFA and healthy controls were represented by the high effect size for glutamate and glutamine measures (above 1.0 for both). Furthermore, using these two plasma measures, we successfully discriminated between the autistic and control groups; the correct classification rate was 91% with a sensitivity of 90% and a specificity of 91%. Therefore, the present results suggest that early assessment of the plasma levels of these two amino acids could be used as a potential screening tool for HFA.

As aforementioned, excess glutamate in extracellular spaces is a potent neurotoxin that leads to neuronal cell death. Perhaps as a result of this, glutamate homeostasis is well maintained by several cell specific elements, including membrane transporters and enzymes that catalyze glutamate metabolism, in both extracellular and intracellular spaces [27]. Glutamate concentrations in plasma are 50–100 µM, whereas, in the whole brain, glutamate concentrations are 10,000–12,000 µM, but they are only 0.5–2 µM in extracellular fluids, indicating that glutamate concentrations are overwhelmingly higher in intracellular spaces [28]. In this concentration gradient, glutamate transporters (excitatory amino acid transporters; EAATs) are thought to play a key role in the periphery as well as in CNS [29]. In the brain capillary endothelial cells, the EAATs exist exclusively in the abluminal membranes and shift glutamate from the extracellular fluids to the endothelial cells. Thus, the EAATs in the abluminal membranes of the blood brain barrier (BBB) act as an active efflux pump for glutamate to reduce the extracellular concentration of glutamate [30]. Furthermore, the EAATs also exist in neurons and astrocytes, where they help to reduce the extracellular glutamate concentration. Recently, several lines of evidence derived from genetic studies have shown the strong association between EAAT3 (also known as SLC1A1, expressed in the CNS and periphery) and autism, suggesting that the EAATs may, in fact, play a part in the pathophysiology of autism [31], [32], [33]. Given that EAATs are altered in the brains of subjects with autism, EAATs may be involved in our finding of plasma glutamate abnormality in autism.

In the cell body, glutamate is regulated by enzymes that catalyze its degradation (e.g., glutamine synthetase [GS], an enzyme that converts glutamate to glutamine) and enzymes that catalyze its biosynthesis (e.g., glutaminase 1 [GLS1], an enzyme that synthesizes glutamate from glutamine and is an isoform dominant in the CNS; and glutamate dehydrogenase 1 [GDH1], an enzyme that synthesizes glutamate from α-ketoglutarate and is an isoform dominant in the brain). In the CNS, GS synthesizes glutamine from glutamate following glial uptake of glutamate in the synaptic cleft. Glutamine is transported back to presynaptic terminals and converted to glutamate by GLS [27]. This multicellular enzymatic process, termed the glutamate-glutamine cycle, is critical for maintaining synaptic function and has been shown to be impaired in various CNS disorders, including epilepsy, Parkinson's disease, Alzheimer's disease, stroke and traumatic brain injury [34]. Since it has been reported that peripheral glutamate/glutamine levels are correlated with those of the CNS [12], [13], our findings of altered plasma levels of glutamate/glutamine are compatible with evidence suggesting the relationship between a dysregulation of glutamine/glutamate metabolism and increased levels of gliosis in the brains of individuals with autism. An increase in gliosis, which is characterized by enhanced activation of astrocytes and microglia, has been observed in the brains of individuals with autism [35], [36]. Interestingly, Ortinski et al. have reported that activated astrocytes downregulate the expression of glutamine synthetase, whereby glutamate is converted into glutamine, which in turn results in reduced glutamine coupled with elevated glutamate [34]. In addition, glutaminase, another enzyme related to glutamate/glutamine metabolism via its conversion of glutamine into glutamate, has been shown to be upregulated in activated microglia [37]. Thus, it is tempting to assume that the process of gliosis generation may be related to the etiology of autism, as mediated by activated astrocytes and/or activated microglia, which may disturb the regulation of certain types of enzymes and thereby alter the metabolism of glutamate/glutamine. Taken together with previous findings, our results demonstrating glutamate/glutamine abnormalities in the plasma of individuals with autism may be indicative of a gliosis process in the autistic brain.

The present results should be interpreted in the context of the following limitations. Since the sample size was small, replication with a larger sample will be needed to confirm our findings. However, the effect size in this study was large (e.g., Cohen's *d* = 1.13 for glutamate), hypothesized increases in the plasma levels of glutamate were supported by the data, and multiple testing was taken into account in the analyses. Thus, the conclusions of the present study can be considered robust. However, to establish plasma glutamate and glutamine levels as screening tools for a diagnosis of autism, further studies using independent samples of children are required. In this study, we investigated a relatively homogeneous subpopulation of individuals with HFA—that is, only drug-naïve, male children with a normal IQ. Thus, the present findings cannot be generalized to the whole population of persons with this disorder. Furthermore, although we focused on a relatively young population of individuals (i.e., children) with HFA in this study, it is of note that each individual received diagnosis early in life and underwent the subsequent course of the disorder for several years. Therefore, it is possible that our findings of higher levels of plasma glutamate and lower levels of plasma glutamine may be attributable to the duration of the condition rather than its pathophysiological mechanism. Nevertheless, the present study showing glutamatergic abnormalities in the peripheral blood of children with autism suggests that these children may have a dysfunction of the brain glutamatergic system. These results provide new insights into the pathophysiology of autism, which may be particularly helpful for the development of novel diagnostic strategies.

## Materials and Methods

### 1. Participants

Twenty-three male children and adolescents with autism who were drug-naïve (age = 13.5±2.5 years [mean ± S.D.]; range = 8–17), and 22 normally developed male controls (age = 12.2±2.4 years; range = 9–17) were recruited for this study (Table 2). The recruitment of persons with autism spectrum disorders was assisted by a nonprofit organization, the Asperger Society of Japan. All participants in both the autism and control groups were Japanese. Enrolled participants with autism were initially assessed by trained child psychiatrists according to the Diagnostic and Statistical Manual of Mental Disorders, Fourth Edition, Text Revision (DSM-IV-TR) [38]. Further, diagnostic evaluation was based on a structured interview with a parent, using the Autism Diagnostic Interview-Revised (ADI-R) [39]. The Wechsler Adult Intelligence Scale, third edition (WAIS-III), or the Wechsler Intelligence Scale for Children, third edition (WISC-III), was employed to evaluate intelligence, and all participants, including those with autism, were confirmed to have a full scale IQ score above 70; i.e., the autism group included only individuals with HFA.

Normally developed children (control group) were recruited through advertisements in local newspapers. All control participants underwent a comprehensive assessment of their medical history to exclude those with neurological or other medical disorders. The Structured Clinical Interview for the DSM-IV Axis-I Diagnoses (SCID-I) was also conducted to screen all participants for any past or present mental illness. Neither neuropsychiatric disorders other than autism nor medical conditions (e.g., epilepsy) that could potentially affect glutamatergic metabolism were found to be present in the 23 participants with HFA. None of the control participants recruited was diagnosed with any neuropsychiatric condition.

All participants and parents were given a complete description of the study, and written informed consent was obtained from all of them prior to enrollment in the study. Approval for the present study was acquired from the ethics committee of the Hamamatsu University School of Medicine.

### 2. Assessment of autistic symptoms

The ADI-R described above was used to provide information on the autism-related symptoms in three separate domains. Domain A quantifies impairment of social interaction (score range: 0–32); domain B evaluates impairment of communication (score range: 0–26); and domain C assesses restricted, repetitive, and stereotyped patterns of behavior and interests (score range: 0–16).

### 3. Blood collection procedure

Plasma samples of the participants in both the HFA and control groups were all collected between 11:00 and 12:00 a.m. before lunch to minimize potential effects of food intake. Blood was taken into 7-ml blood collection tubes containing EDTA-2Na, and samples were immediately centrifuged at 1000×g for 15 minutes to obtain platelet-poor plasma. The plasma was decanted, aliquoted to avoid multiple freeze-thaw cycles and stored for up to 4 months at −80°C until analysis.

### 4. Amino acid measurement

The plasma samples obtained were homogenized in 1.5 volumes of 5% 5-sulfosalicylic acid (final concentration: 2.0%). The homogenates were centrifuged immediately at 12000 rpm 4°C for 10 minutes to remove precipitated protein. The supernatants were collected and used for amino acid measurement. The concentration of amino acids in the plasma samples was measured using an automatic HPLC system (L-8500A; Hitachi High-Technologies Corporation, Tokyo, Japan). Briefly, amino acids, separated by cation-exchange chromatography, were detected spectrophotometrically after postcolumn reaction with ninhydrin reagent. Although we examined 41 amino acids, 16 of them were undetectable in the plasma using HPLC. Consequently, the 25 amino acids examined were as follows: alanine, α-aminobutyric acid, arginine, asparagine, citrulline, cystine, glutamate, glutamine, glycine, histidine, hydroxyproline, isoleucine, leucine, lysine, methionine, ornithine, phenylalanine, proline, serine, taurine, threonine, tryptophan, tyrosine, urea, and valine (see Table 1). The 16 amino acids that were unmeasurable by HPLC in either the autistic or control group were as follows: ß-alanine, α-aminoadipic acid, γ-aminobutyric acid, γ-amino ß-hydroxybutyric acid, ß-amino-iso-butyric acid, anserine, aspartic acid, carnosine, cystathionine, homocystine, hydroxylysine, 1-methylhistidine, 3-methylhistidine, monoethanolamine, phosphoethanolamine, and sarcosine. The plasma creatinine level, which reflects the quantity of muscle mass, was also measured using the creatininase-creatinase-sarcosine oxidase-POD method [40]. This assessment was made because the skeletal muscle accounts for a major portion of glutamine synthesis in the human body; thus, varying amounts of muscle mass in individuals could have a confounding effect, especially when amino acid levels of glutamate and glutamine are evaluated.

### 5. Statistical analysis

All data are presented as the mean ± standard deviation (S.D.), unless otherwise indicated. The data were analyzed using the unpaired *t*-test due to the approximate normal distribution of the data. The relationships between amino acid levels and clinical variables among individuals with autism were evaluated by Pearson's correlation coefficients. As we examined each of the 25 amino acids levels separately, a *p*-value of less than 0.002 was considered to be statistically significant, according to the Bonferroni correction [41]. Analysis of covariance (ANCOVA) was used to allow for covariates. We calculated the effect size (Cohen's *d*) for variables with a significant group difference.

Then, we investigated whether amino acids could serve as a diagnostic tool using a discriminant function analysis. In the logistic regression analysis, we calculated the correct classification rate. All analyses were performed with Stata/SE 10.0 software for windows (Stata Corp., College Station, TX).
